# Is Stem Revision Necessary for Vancouver B2 Periprosthetic Hip Fractures? Analysis of Osteosynthesis Results from 39 Cases

**DOI:** 10.3390/jcm10225288

**Published:** 2021-11-14

**Authors:** David González-Martín, Sergio González-Casamayor, Mario Herrera-Pérez, Ayron Guerra-Ferraz, Jorge Ojeda-Jiménez, José Luis Pais-Brito

**Affiliations:** Orthopedic Surgery and Traumatology Service, Hospital Universitario de Canarias, Universidad de La Laguna, Carretera Ofra S/N, 38320 San Cristóbal de La Laguna, Santa Cruz de Tenerife, Spain; davidglezmartin@gmail.com (D.G.-M.); sgcasamayor@gmail.com (S.G.-C.); ayronguerra@gmail.com (A.G.-F.); jojedajim@gmail.com (J.O.-J.); paisbrito@gmail.com (J.L.P.-B.)

**Keywords:** periprosthetic fracture, hip, osteosynthesis, Vancouver, B2

## Abstract

Although stem revision is recommended for Vancouver B2 periprosthetic hip fractures (PPHFs), there has recently been a debate whether, under certain conditions, they could be treated by osteosynthesis alone. This study aimed to describe the medium-term clinical and radiological results of several patients with V-B2 fractures treated via osteosynthesis. A retrospective study of patients with V-B2 PPHF treated by osteosynthesis without stem revision, operated on between 2009 and 2019, was performed. The type of arthroplasty, type of stem, ASA, Charlson Comorbidity Index (CCI), medical and implant complications, reoperation rate, first-year mortality, radiological results (consolidation time), and functional results were analyzed. Thirty-nine patients were included. Their average age was 78.82 years. Most of the patients presented ASA ≥ 3 (35/39) and CCI ≥ 5 (32/39). Radiological consolidation was achieved in 93.5% of patients, with an average consolidation time of 92.93 days. The average Parker test score before admission was 5.84 while the current one was 4.92 (5.16 years follow-up). Osteosynthesis without stem revision is a valid surgical alternative in certain types of patients with V-B2 PPHF, depending on previous mobility, fracture pattern (anatomical reconstruction possible), anesthetic risk, comorbidities, and previous hip pain.

## 1. Introduction

Periprosthetic hip fractures (PPHFs) are becoming increasingly common due to the ageing population and the rise in the number of hip replacements [1].

The Vancouver classification [2] is the most used for managing patients with PPHF [3]. According to the authors, type B2 fractures require stem revision using a longer stem. However, several authors have argued that under certain conditions, type B2 fractures could be successfully treated with the osteosynthesis technique [3,4,5,6,7,8,9,10,11].

This study aimed to describe the results achieved at our institution in patients with Vancouver B2 (V-B2) PPHF treated with osteosynthesis, by examining the consolidation rate, implant, and medical complications, as well as medium-term functional outcomes.

## 2. Materials and Methods

Institutional review board approval was obtained before the initiation of this study. A revision observational study was conducted, including all patients operated on between 2009 and 2019 who had a diagnosis of PPHF.

The preoperative X-rays were independently reviewed by four of the authors to check that the Vancouver Classification [2] was applied accurately on admission. Intraoperative stem stability was collected to determine that it was indeed a Vancouver type B2. The inclusion criterion was PPHF V-B2 treated through osteosynthesis without stem revision. Fractures of Vancouver types A, B1, B3, C, and those B2 fractures treated either conservatively or by stem revision were excluded.

The type of arthroplasty (hemiarthroplasty/hip replacement), type of stem (cemented/non-cemented; straight/anatomic), hospital stay, medical and implant complications, reoperation rate, first-year mortality, radiological results (consolidation time), and functional results were analyzed. All those patients who had undergone surgery with at least one year of follow-up were reviewed in the hospital. Most patients came for consultation, but for those who could not (dementia, non-ambulation, etc.) the main caregiver was contacted. The pre-fracture Parker mobility test (PMT) [12] was collected from the discharge report and the current PMT and Oxford Hip Score [13] were collected at the current consultation.

### 2.1. Surgical Technique:

VB2 PPHF was decided based on previous mobility, whether the fracture pattern made anatomical reconstruction possible, anesthetic risk, and previous hip pain. The main factor to decide was previous mobility, comorbidities (Charlson Comorbidity Index—CCI) [14], and anesthetic risk (ASA) [15]. In elderly and multi-pathological patients (ASA ≥ 3 and/or CCI ≥ 5), osteosynthesis was chosen, while in young patients with high functional demand, stem revision was preferred.

**Figure 1:**
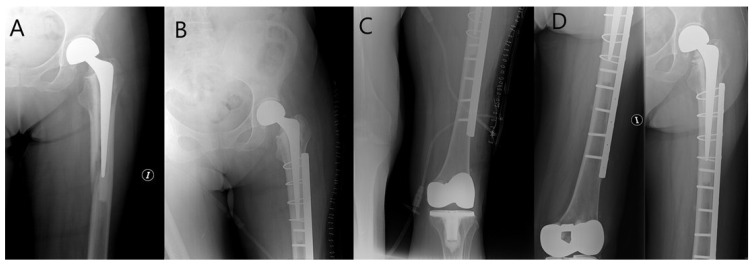
Woman 77 years old, Vancouver B2 PPHF treated using ORIF. (**A**) Fracture; (**B**,**C**) postoperative control; (**D**) 4-year control.

**Figure 2:**
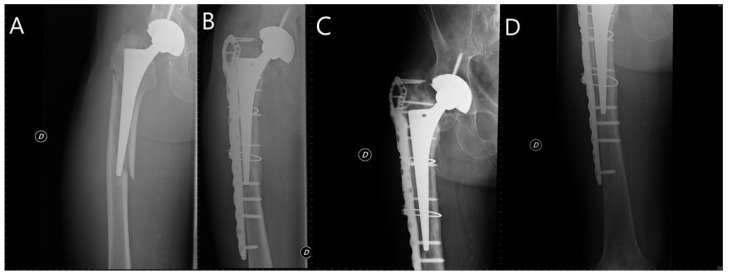
Woman 77 years-old, Vancouver B2 PPHF treated using ORIF. (**A**) Fracture; (**B**) 6-month control (consolidated fracture); (**C**,**D**) 18-month control.

Osteosynthesis technique (Figure 1, Figure 2): the patient was placed in the lateral decubitus position if it was necessary to change the decision to stem revision. Without performing a hip arthrotomy and with a lateral subvastus approach, the fracture line was reached. Then, the main surgeon, by direct vision or palpation of the fracture site, asked the assistant to perform distal external and internal rotation to assess whether any of the main fragments were still attached to the stem. In that case, osteosynthesis was performed. If we found a large fragment attached to the stem and osteosynthesis was possible, we performed it. Otherwise, we performed a stem revision. In some patients with a completely loose stem but multi-pathological and low functional demand, osteosynthesis was also decided. Postoperative management: patients remained without weight-bearing for 6–8 weeks and later progressed from partial until full weight-bearing according to the pain.

Current control radiographs were reviewed to assess fracture healing (bone bridge in at least 3 of the 4 cortices), stem subsidence, loosening (Harris criteria) [16], malunion (>5° in the frontal and sagittal plane), and femoral osteolysis.

### 2.2. Statistical Analysis

Proportional comparisons were made using chi-square or Fisher’s exact tests, as appropriate. All analyses were carried out using SPSS v.25 package (IBM Corp. Released 2017. IBM SPSS Statistics for Windows, Version 25.0 Armonk, NY, USA). The result was considered significant when the *p*-value was less than 0.05.

## 3. Results

Between 2009 and 2019, 39 patients were included. Their mean age was 78.82 years (R 45–92). There were 25 females and 14 males. The mean total follow-up time was 3.2 years (1174 days (R 26–3145; SD 781)). The side most affected was the right side (59%). The type of arthroplasty consisted of 4 hemiarthroplasties and 35 hip replacements, the type of stem consisted of 9 cemented and 30 non-cemented, 29 straight, and 10 anatomic. The average surgery time was 104 min (R 40–175). The average total hospital stay was 24.51 days (R 10–89). A total of 34 patients were given implanted plates, with or without associated cerclage while 5 patients received only cerclage.

Radiological consolidation was achieved in 93.5% of patients (29/31) (eight were lost because they missed successive postoperative appointments), with an average consolidation time of 92.93 days (R 40–166) (Table 1). The consolidation curve shows a 94-day mean consolidation (R 83.9–104.1; SD 5.16). In two patients, no consolidation was detected; both patients presented fractures with multiple fragments that were difficult to synthesize, but due to the low functional demand and high anesthetic risk, the decision was made to apply osteosynthesis instead of making a stem revision: one was non-ambulatory and another was clinically well tolerated.

In the current assessment, after discovering that 13 patients had died (33.3%) and 1 patient was missing, 25 patients were analyzed, with an average follow-up time of 1883 days (5.16 years) (R 556–3811; SD 980). The average PMT before admission was 5.84, while the average PMT in the current review was 4.92, showing a loss of about 1 point. The current mean Oxford Hip Score, which ranges from 0 as the worst value to 48 being the best possible value, was 26.76 (R 10–41; SD 7.3).

There were five implant complications (four loosening and one varus consolidation) (Table 2). All of them occurred in uncemented straight stems, but this difference was not significant when compared to anatomical or cemented stems. Of those, two required a stem revision due to clinical instability and persistent pain (1.5 and 2 years after fracture surgery, respectively), while the other three were treated conservatively due to acceptable clinical tolerance. Seven patients suffered medical complications, including four deep wound infections requiring lavage and debridement without removing osteosynthesis. The need for reoperation including all causes was therefore 15.3% (6/39). The mortality rate in the first year was 15.4% (6/39).

## 4. Discussion

The Vancouver classification [2] is currently the most widely used in the management of PPHF. This takes into account the fracture location, implant stability, and bone stock. It has been validated in cemented stems [17] but has not been able to demonstrate the same validity in uncemented stems [18]. The authors define that an arthroplasty revision should be performed, with a longer stem, despite good bone stock [2,19].

Several studies have been published reporting good results in VB2 PPHF treated with osteosynthesis [4,5,11] and some of their authors have argued that the Vancouver classification, although very useful, should include the patient’s previous functionality, the anesthetic risk, comorbidities, the possibility of achieving anatomical reduction, the stability of the cement mantle, age, and the surgeon’s experience in deciding what type of treatment to perform [5,8,9,11]. In our hospital, the decision on whether to perform osteosynthesis or stem revision is made based on the Vancouver classification, previous mobility, whether the fracture pattern [20] made anatomical reconstruction possible, anesthetic risk, comorbidities, previous hip pain, and the experience of the senior orthopedic surgeons.

Stoffel et al. [3] conducted a systematic review of osteosynthesis vs. revision arthroplasty in cases of loosened stems (V-B2/B3) and reported that, in certain cases, osteosynthesis can deliver equivalent or even better results [1,3,4,5,6,7,8,9,10,11,21]. On the other hand, Khan et al. [22] in another review on the same topic showed that osteosynthesis alone is associated with a higher percentage of reoperation rate, but the relative risk did not reach a statistically significant value [22].

Several articles have shown good results in V-B2 fractures around cemented polished tapered stems treated by osteosynthesis [4,5,23]. In the present study, all the implant complications occurred around uncemented straight stems, but this difference was not significant when compared to anatomical or cemented stems. Most of the cemented stems were cemented polished tapered femoral stems, with no complications occurring. Among the cementless stems, there was a diversity of designs (fully coated, tapered rectangle, tapered round, etc.), without finding differences between them.

Most studies show poor functional outcomes in this type of patient, often due to the previous baseline situation, whatever the chosen treatment [10,24]. However, some authors show better functional results [25]. Although the vast majority of fractures heal, few patients achieve functional results similar to those previously achieved [24,26]. In our sample, 93.5% of our fractures healed within an average of 92.93 days, similar to other previously reported on type B PPHF [6].

The vast majority of patients with PPHF are older, fragile, and have many comorbidities in addition to poor bone quality [10]. For this reason, most studies report high rates of medical and surgery-related complications, reoperation, and mortality in the first year. Zuurmond et al. analyzed 71 PPHF including all Vancouver types and found 31% medical complications (both minor and major), 9.8% mortality in the first year, and 32% reoperation from whatever cause (refracture, dislocation, infection, etc.) [10]. On the other hand, Joestl et al. found that in a study of 36 patients with V-B2 fractures, of which 8 were treated by ORIF and the rest by revision, 14% had complications, all of whom were in the revision group (three dislocations and two infections) [8]. Füchtmeier et al. published a study of 121 PPHFs, of all Vancouver types, treated according to the most widely accepted algorithm [4], reporting a reintervention rate of 17.3% and a mortality rate of 13.2% in the first year [27]. The results of this latter study are very similar to ours: 17.9% medical complications (wound infection, pneumonia, etc.); 12.8% implant complications (four prosthetic loosenings and one varus consolidation). The surgical reoperation rate in our study was 15.3% (6/39), as four deep wound infections and two loosenings were not tolerated and replacements were given. Our first-year mortality rate was 15.9% (6/39), similar to that described in other studies of PPHF, which report first-year mortality rates of 3.3–34% [9,10,27].

It is widely accepted that patients with PPHF suffer from a generalized functional decline and decreased mobility [1]. In our sample, the PMT before surgery was 5.84 and the current one, after an average follow-up of 5 years, was 4.92, showing an average loss of almost 1 point on this scale. This result is very similar to that from another study of ORIF performed on Vancouver B fracture patients [6]. In that study, 32% (8/25) maintained the same PMT, 48% (12/25) decreased one point, 16% (4/25) went down 2 points, and 4% (1/25) dropped 3 points in this mobility test. This means that 68% of patients had experienced functional worsening at 5 years after surgery.

There are few papers in the literature that have statistically analyzed the functional outcomes in V-B2 patients, comparing the ORIF with stem revision. Solomon et al. found no significant differences in either the Harris Hip Score (HHS) or mobility [4], and Joestl et al. reported nonsignificant but favorable differences concerning ORIF regarding the number of patients who returned to their previous mobility levels [8]. On the other hand, Gitajn et al. published results in favor of revision due to early weight-bearing authorization in this group [21]. Our analysis did not include a control group of stem revision, but we did register a general functional worsening as described in other studies, for example, the study of Lindahl et al. included 321 periprosthetic fractures and although they did not obtain the HHS before the fracture, they did demonstrate low scores in the postoperative HHS, and patient quality of life was lower than the quality of life following primary hip prosthesis [1].

The use of cerclages in isolation is insufficient osteosynthesis to treat PPHF [28]. In our sample, this treatment was applied in five multi-pathological patients, bedridden, with very low functional demand and minimally displaced fractures, but we fully agree that to achieve sufficient stability, it is necessary to use a plate and sufficient proximal and distal screws as expressed by other authors [9,28].

Preoperative stay in our sample was very long (8.85 days) and we know that it is not representative of most hospitals. It depends on many factors, relating to both the patient (optimization before surgery) and hospital logistics (availability of the operating theatre, availability of surgeons, other emergencies, etc.). Several studies state that delayed surgery increases the mortality rate, setting the limit between 2 and 5 days [9]. The utmost effort should be made to reduce this time and thereby optimize the results.

The weakness of our study is its retrospective nature, the lack of a control group, the loss of patients to follow-up (1/3 died) and the majority of patients were elderly.

## 5. Conclusions

The results of our study show that osteosynthesis is a valid surgical alternative in certain types of patients with V-B2 PPHF. This option necessitates a comprehensive study of the patient, which takes into account the Vancouver classification, prior mobility, functional demand, anesthetic risk, fracture pattern (anatomical reconstruction possible), previous pain in the hip, and the experience of the senior orthopedic surgeons. However, prospective multicenter studies should be carried out to obtain a greater level of evidence.

## Figures and Tables

**Table 1:** Medium-long term assessment. * Current review: 25 patients (13 deaths and 1 follow-up loss).

Medium to Long-Term Follow-up	
1 year (*n*= 39)	
Death within the first year	15.4% (6/39)
Radiological consolidation	Yes 29 (93.5%); No 2 (6.5%) (8 losses)
Consolidation time	92.93 days (R 40–166; SD 30.4)
3 years (*n* = 39)	
Average follow-up time	1174 (3.2 years) (R 26–3145; SD 781)
Implant complications	12.8% (5/39)
Patient complications	17.9% (7/39)
Need for reintervention	15.3% (6/39) - Convert ORIF → Revision (5.1% (2/39)) - Lavage and debridement (10.2% (4/39))
5 years (*n* = 25) *	
Average follow-up time	1883 days (5.16 years) (R 556–3811; SD 980)
Death in the current review	33.3% (13/39)
Prior Parker mobility test	5.84 (R 0–9; SD 2)
Current Parker mobility test	4.92 (R 0–9; SD 2)
Current OHS	26.76 (R 10–41; SD 7.3)

**Table 2:** Patients with Vancouver type B2 PPHF treated using osteosynthesis. (F = female, M = male, Impl. = Implant, HA = hemiarthroplasty, HR = hip replacement, Reinterv. = need for reintervention).

N	Age	Sex	Impl.	Implanted material	Implant Complication	Medical complication	Reinterv.
1	68	F	HR	NCB plate (Zimmer)	Loosening		Yes
2	72	M	HR	NCB plate (Zimmer)			No
3	78	F	HR	NCB plate (Zimmer)			No
4	90	F	HR	NCB plate (Zimmer)			No
5	81	F	HR	NCB plate (Zimmer)			No
6	89	F	HR	NCB plate (Zimmer)			No
7	73	F	HR	NCB plate (Zimmer)			No
8	84	M	HR	NCB plate (Zimmer)			No
9	85	F	HR	NCB plate (Zimmer)			No
10	83	F	HR	NCB plate (Zimmer)	Loosening		No
11	85	F	HR	NCB plate (Zimmer)		Wound infection	Yes
12	84	F	HR	NCB plate (Zimmer)	Malunion		No
13	89	F	HR	NCB plate (Zimmer)			No
14	92	M	HR	NCB plate (Zimmer)			No
15	86	M	HR	NCB plate (Zimmer)		Acute cholecystitis	No
16	84	F	HR	NCB plate (Zimmer)		Wound infection	No
17	87	M	HR	NCB plate (Zimmer)		Pneumonia	No
18	88	M	HA	NCB plate (Zimmer)			No
19	80	F	HR	NCB plate (Zimmer)			No
20	82	F	HA	NCB plate (Zimmer)		Wound infection	Yes
21	45	M	HR	NCB plate (Zimmer)	Loosening		No
22	59	M	HR	NCB plate (Zimmer)			No
23	73	F	HR	Double LCP plate (Synthes)			No
24	86	F	HR	LCP plate (Synthes)			No
25	84	M	HR	LCP plate (Synthes)			No
26	80	F	HR	LCP plate (Synthes)			No
27	60	M	HR	Dall-Miles plate (Stryker)	Loosening		Yes
28	89	F	HA	Dall-Miles plate (Stryker)			No
29	67	F	HR	Cable-ready plate (Zimmer)		Wound infection	Yes
30	84	F	HR	Cable-ready plate (Zimmer)			No
31	78	M	HR	Cable-ready plate (Zimmer)			No
32	63	F	HR	Cable-ready plate (Zimmer)		Pneumonia	Yes
33	80	F	HR	Cable-ready plate (Zimmer)			No
34	77	F	HA	Cable-ready plate (Zimmer)			No
35	90	M	HR	Cerclage			No
36	74	M	HR	Cerclage			No
37	77	F	HR	Cerclage			No
38	83	F	HR	Cerclage			No
39	65	M	HR	Cerclage			No
